# Effect of Cu species on N_2_O formation in the NH_3_-SCR reaction over Cu-SSZ-13

**DOI:** 10.1039/d6ra04872b

**Published:** 2026-08-03

**Authors:** Feng Feng, Xiankun Wang, Jiangli Ma, Hongyao Luo, Han Zhao, Dongxia Yang, Kongzhai Li, Hua Wang

**Affiliations:** a Kunming University of Science and Technology, School of Metallurgical and Energy Engineering Kunming China youxiang11662026@126.com +86-0871-6839-3370; b Kunming Sino-Platinum Metals Catalyst Co, Ltd Kunming China; c State Key Laboratory of Precious Metal Functional Materials Kunming China

## Abstract

This work systematically investigates the effect of Cu/Al ratio on NO_*x*_ conversion, N_2_O formation, NH_3_ oxidation, and hydrothermal stability over Cu-SSZ-13 catalysts under simulated diesel-exhaust NH_3_-SCR conditions. Multiple characterization techniques, including XRD, H_2_-TPR, NH_3_-TPD, UV-vis DRS, XPS, EPR, and *in situ* DRIFTS, were employed to clarify the relationship between Cu-species evolution, surface intermediate reactivity, and N_2_O formation. The results show that the Cu/Al ratio strongly regulates the distribution of Z[Cu^2+^(OH)]^+^, Z_2_Cu^2+^, and CuO_*x*_-like species. An insufficient Cu loading limits the number of redox-active Cu species required for low-temperature SCR, whereas excessive Cu loading promotes the formation of CuO_*x*_-like species and less selective Cu environments, leading to enhanced NH_3_ oxidation and N_2_O formation. After hydrothermal aging at 800 °C for 16 h, dealumination of the zeolite framework disrupts the coordination bonds between active Cu^2+^ and framework Al, driving the transformation to CuO_*x*_. When the Cu/Al ratio exceeds 0.3, it further generates inert CuAlO_*x*_ that lack SCR activity and strongly promote N_2_O formation. Since low-temperature N_2_O formation is closely associated with the formation and decomposition of NH_4_NO_3_-related intermediates, these results indicate that N_2_O suppression over Cu-SSZ-13 is not governed simply by Cu loading, but by maintaining an appropriate relative distribution of Cu species.

## Abstract 2

This work systematically investigates the effect of Cu/Al ratio on NO_*x*_ conversion, N_2_O formation, NH_3_ oxidation, and hydrothermal stability over Cu-SSZ-13 catalysts under simulated diesel-exhaust NH_3_-SCR conditions.

## Graphical abstract

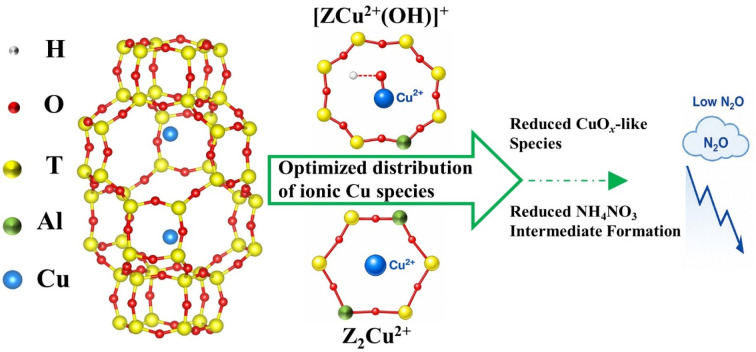

## 1 Introduction

The escalating global climate crisis, primarily driven by fossil fuel combustion, has intensified focus on low-carbon and even zero-carbon energy systems.^1^ Among key emission sources, the transportation sector plays a pivotal role, accounting for approximately 25% of global carbon emissions according to the International Energy Agency (IEA) data.^2^ This underscores the urgent need for transformative policies and technological innovations to decarbonize transportation and align with climate mitigation goals. In response, governments worldwide have implemented increasingly stringent emission regulations. A prominent example is Regulation (EU) 2024/1257 (Euro 7), which tightens permissible nitrogen oxides (NO_*x*_) emissions by 56.5% compared to Euro 6 and introduces new limits for nitrous oxide (N_2_O) emissions.^3^ These measures reflect the growing emphasis on curbing pollutants that worsen air quality and contribute to climate change. These requirements highlight the urgent need for after treatment technologies that can simultaneously achieve efficient NO_*x*_ removal and suppress N_2_O formation. Under lean-burn diesel exhaust conditions, selective catalytic reduction of NO_*x*_ with NH_3_ (NH_3_-SCR) is the most widely used technology for NO_*x*_ abatement. Since NO generally constitutes the major fraction of NO_*x*_ in diesel exhaust, the standard SCR reaction (R1) is usually the dominant pathway; when a portion of NO is oxidized to NO_2_ over an upstream oxidation catalyst, NO and NO_2_ can jointly participate in the fast SCR reaction (R2); under NO_2_-rich conditions, the NO_2_-SCR reaction (R3) may also occur; in addition to these desired NO_*x*_ reduction pathways, competing side reactions (R4)–(R6) can take place, especially at elevated temperatures or over non-selective oxidative sites.

R1 4NH_3_ + 4NO + O_2_ → 4N_2_ + 6H_2_O

R2 2NH_3_ + NO + NO_2_ → 2N_2_ + 3H_2_O

R3 8NH_3_ + 6NO_2_ → 7N_2_ + 12H_2_O

R4 4NH_3_ + 3O_2_ → 2N_2_ + 6H_2_O

R5 2NH_3_ + 2O_2_ → N_2_O + 3H_2_O

R6 4NH_3_ + 4NO + 3O_2_ → 4N_2_O + 6H_2_O

To comply with stringent NO_*x*_ emission regulations, dual-stage NH_3_-SCR systems based on commercial Cu-based zeolite (Cu-SSZ-13 or Cu-SSZ-39) have been widely integrated into the after-treatment system (ATS) of diesel engines. However, the implementation of this advanced ATS has inadvertently led to secondary N_2_O emissions, as highlighted in previous studies.^4–6^ Numerous studies have extensively documented the formation pathways of N_2_O over Cu-based zeolite catalysts, reporting the influence of reaction temperature, gas compositions (NO_2_, O_2_, NO, NH_3_, H_2_O, and sulfur), zeolite framework and aging treatment. Notably, studies reveal a distinct “double-peak” characteristic in N_2_O generation with increasing temperature, suggesting different formation mechanisms at low-temperature (LT) and high-temperature (HT) ranges.^7^ The temperature-dependent N_2_O formation mechanisms remain debated. Some researchers^8^ have found that LT-pathways may involve either direct NH_3_–NO/NO_2_ reactions or intermediate decomposition, though the exact copper species and their locations remain contested. Olsson *et al.*^9^ attribute LT N_2_O formation to the oxidation of Cu–NH_3_–NO precursors, whereas Daya,^10^ Gao,^11^ and Yu^12^ implicate NH_4_NO_3_ decomposition. HT-mechanisms include direct NH_3_ oxidation and non-selective NH_3_-NO_*x*_ reactions, though the details still require further elucidation.^13^ Chen *et al.*^14^ proposed that N_2_O formation primarily originates from NO oxidation to NO_2,_ followed by subsequent surface intermediate reactions, highlighting the critical role of NO_2_ concentration. Reaction kinetics further demonstrate that while N_2_O formation shows zero-order dependence on gaseous NO concentration (*i.e.*, unaffected by NO concentration), it exhibits a positive correlation with NH_3_ concentration.^15^ Notably, O_2_ in diesel exhaust influences N_2_O formation through different redox pathways, as evidenced by its distinct impact pattern compared to conventional SCR behavior.^16^ H_2_O exerts dual effects, competing with NH_3_ for active sites and enhancing surface acidity by increasing –OH groups, ultimately modifying SCR selectivity.^16,17^ Sulfur poisoning introduces additional complexity through sulfate formation, which modifies active sites and intermediate chemistry.^18^ This discrepancy stems from both structural and redox properties.

Despite extensive studies on reaction atmospheres and influencing factors, a deeper understanding of Cu-SSZ-13 zeolite itself is necessary. Cu-SSZ-13 is a representative Cu-exchanged small-pore zeolite with the chabazite (CHA) topology, in which large CHA cages are connected through eight-membered-ring (8MR) windows with an aperture of approximately 3.8 Å. Such a confined microporous structure contributes to the hydrocarbon tolerance and hydrothermal stability of Cu-SSZ-13, while also affecting the diffusion, accumulation, and transformation of surface intermediates involved in N_2_O formation. Catalyst aging further modulates N_2_O generation through structural evolution and active site reconstruction. Therefore, it is essential to thoroughly investigate the evolution of copper species during hydrothermal aging, aiming to develop strategies for suppressing N_2_O formation from the perspective of the zeolite framework. Aa a baseline, for fresh Cu-SSZ-13, it has been reported that Cu mainly exists as Z_2_Cu^2+^ located on six-membered rings (6 MR) and as Z[Cu^2+^(OH)]^+^ situated in CHA cages or near eight-membered rings (8 MR).^19^ The proportion of Z_2_Cu^2+^ and Z[Cu^2+^(OH)]^+^ is co-regulated by the framework and Cu loading. During the SCR reaction, Z_2_Cu^2+^ dominates the catalytic activity in the HT range and exhibits lower N_2_O selectivity.^20^ In contrast, Z[Cu^2+^(OH)]^+^ dominates the activity in the LT range but, due to its strong oxidizing ability, tends to promote the decomposition of NH_4_NO_3_ to form N_2_O.^21^ Furthermore, excessively high Cu loading leads to the formation of CuO_*x*_ clusters, which further aggravate non-selective NH_3_ oxidation and increase the formation of the byproduct N_2_O.^22^ When catalysts are hydrothermally aged, Z_2_Cu^2+^ species show excellent stability, and their content remains largely unchanged even under relatively harsh aging conditions.^23^ Conversely, Z[Cu^2+^(OH)]^+^ species are more sensitive and prone to migration and transformation, and they partially convert to Z_2_Cu^2+^ or aggregate into CuO_*x*_ clusters even under relatively mild hydrothermal aging conditions.^23,24^

Although the influence of Cu loading on the NH_3_-SCR activity and hydrothermal stability of Cu-SSZ-13 has been widely investigated, the relationship between Cu-species evolution and N_2_O formation is still not fully clarified, especially from the perspective of surface intermediate reactivity.^25–28^ Previous studies have mainly focused on the distribution of Cu species, overall NO_*x*_ conversion, or hydrothermal deactivation behavior, whereas direct evidence connecting Cu/Al-regulated Cu species, hydrothermal-aging-induced Cu redistribution, and the formation/consumption of NH_3_/NO_*x*_-derived intermediates remains limited. In particular, the roles of Z[Cu^2+^(OH)]^+^, Z_2_Cu^2+^, and CuO_*x*_-like species in regulating low-temperature N_2_O formation require further clarification.^20,28^ Therefore, in this work, Cu-SSZ-13 catalysts with different Cu/Al ratios were systematically investigated before and after hydrothermal aging in terms of their performance, structure, acidity, redox properties, and surface reactions. By correlating Cu-species evolution with NH_3_/NO_*x*_ intermediate reactivity and N_2_O formation, this study aims to provide a clearer mechanistic understanding of how Cu/Al regulation affects SCR selectivity and N_2_O suppression over Cu-SSZ-13 catalysts.

## 2 Experimental

### 2.1 Preparation of Cu-SSZ-13 zeolites

Cu-SSZ-13 was synthesized *via* hydrothermal crystallization followed by copper ion exchange.^29^ The SiO_2_/Al_2_O_3_ ratio of 21 was controlled by adjusting the molar composition of the initial gel. 1 g of as-made Na-SSZ-13 was added to 20 mL of 1 mol per L NH_4_HCO_3_ solution, and the mixture was stirred vigorously at 80 °C for 5 h. After the ammonium exchange, the product was washed with deionized water until the pH reached neutral. The filtered cake was dried at 100 °C overnight and then calcined at 600 °C for 3 h to obtain H-SSZ-13. Cu-SSZ-13 samples with different Cu loadings were prepared using Cu(CH_3_COO)_2_·H_2_O as the copper source. Specifically, 1.0 g of H-SSZ-13 was dispersed in 20 mL of Cu(CH_3_COO)_2_·H_2_O aqueous solutions with concentrations of 0.02, 0.03, 0.04, and 0.05 mol L^−1^, respectively. The suspensions were vigorously stirred at 80 °C for 5 h to obtain samples with different Cu/Al ratios. Once the Cu exchange was completed, the samples were washed until the pH reached neutral. The filtered cake was dried in an oven at 100 °C overnight and then calcined in a muffle furnace at 600 °C for 3 h to produce the Cu-SSZ-13 powder. The actual Cu/Al ratios reported in this work were calculated from the Cu contents determined by ICP-OES and the Al contents derived from XRF analysis. The actual Cu contents and Cu/Al ratios of all samples are summarized in Table 1. The four Cu-SSZ-13 samples with controlled Cu/Al ratios were named as Cu/Al-1-F(Cu/Al = 0.12), Cu/Al-2-F(Cu/Al = 0.25), Cu/Al-3-F(Cu/Al = 0.35), Cu/Al-4-F(Cu/Al = 0.45), where the suffixes “F” denotes the fresh states.

**Table 1:** Physicochemical properties of the prepared samples

Sample	SiO_2_/Al_2_O_3_^a^	Cu/Al	Cu b(%)	BET (m^2^ g^−1^)^c^	Pore volume d(cm^3^ g^−1^)	Pore diameter (nm)^e^
Cu/Al-1-F	21.1	0.12	1.09	606	0.29	0.36
Cu/Al-2-F	20.9	0.25	2.26	600	0.30	0.36
Cu/Al-3-F	21.5	0.35	3.11	593	0.31	0.37
Cu/Al-4-F	21.2	0.45	3.98	572	0.28	0.36
Cu/Al-1-A	21.1	0.12	1.09	588	0.29	0.35
Cu/Al-2-A	20.9	0.25	2.26	576	0.30	0.35
Cu/Al-3-A	21.5	0.35	3.11	567	0.30	0.34
Cu/Al-4-A	21.2	0.45	3.98	543	0.25	0.29

a Calculated by the XRF results.

b Determined by the ICP-OES analyzer.

c Calculated by the BET method.

d Calculated by the *t*-plot method.

e Calculated by the BJH method.

### 2.2 Hydrothermal aging of Cu-SSZ-13 zeolites

Samples were subjected to accelerated aging at 800 °C for 16 h in a quartz tube furnace with a gas composition of 7% H_2_O and 93% air, maintained at a space velocity of 240 000 h^−1^. The obtained samples were named as Cu/Al-*X*-A (*X* = 1, 2, 3, and 4), where the suffixes “A” denotes the hydrothermally aged states.

### 2.3 Material characterization

The Cu contents of the prepared samples were quantified using a PerkinElmer ICP 2100 optical emission spectrometer after dissolving the solids in a mixture of HNO_3_, HCl and HF.

The SiO_2_ and Al_2_O_3_ contents of the prepared samples were measured using a PANalytical Axios MAX XRF spectrometer.

The specific surface area and pore size of the prepared samples were analyzed by N_2_ adsorption–desorption isotherms using a Quantachrome NovaWin2 instrument. Before testing, the sample were pretreated under vacuum at 150 °C for 2 h. The specific surface area was calculated using the Brunauer–Emmett–Teller (BET) method, while the pore volume and pore size distribution were determined employing the *t*-plot method and BJH method, respectively.

Powder X-ray diffraction (PXRD) measurements were conducted on a Philips X'pert Pro diffractometer equipped with a graphite monochromator and a multi-sample stage. The instrument was operated at 40 kV and 40 mA, scanning from 10° to 90° (2*θ*) at a rate of 10° min^−1^, using Cu Kα radiation (*λ* = 0.15418 nm).

A UV-visible-near-infrared (UV-vis-NIR) diffuse reflectance spectrometer (DRS) was performed using a Shimadzu UV-2600 spectrometer to analyze the structural changes of the prepared samples. The measurements were carried out with the spectrometer equipped with an integrating sphere, using BaSO_4_ as a reference. The scanning range was from 7500 cm^−1^ to 50 000 cm^−1^.

The morphology of the as-prepared samples was studied by scanning electron microscopy (SEM) using a JEOL JSM-7800F microscope and by high-resolution transmission electron microscopy (HRTEM) using a TECNAI F-30 microscope operated at an accelerating voltage of 300 kV.

X-ray photoelectron spectroscopy (XPS) was employed to identify the chemical states of Cu species on Cu-SSZ-13 using a K-Alpha^+^ spectroscope (Thermo Fisher Scientific, USA) equipped with a monochromatic Al Kα source (1486.6 eV) operated at 6 mA and 12 kV. The binding energy was calibrated using the surface contamination C 1s peak at 284.8 eV as a reference.

The NH_3_ temperature-programmed desorption (NH_3_-TPD) test was carried out on a CHEMBET3000 instrument. 100 mg of the as-prepared sample was loaded into a quartz U-tube reactor, then an ammonia flow was fed at 80 °C for 1 h until the thermal conductivity detector (TCD) signal stabilized. Afterwards, the feed gas was switched to He to flush the adsorbed NH_3_ from the catalyst surface. After 1 h, NH_3_-TPD was performed, with the temperature rising from 50 to 800 °C at a rate of 10 °C min^−1^ under a He stream.

H_2_ temperature-programmed reduction (H_2_-TPR) was carried out on the CHEMBET3000 instrument. 100 mg of the as-prepared sample was loaded into a quartz U-tube reactor and then pretreated under Ar flow at 300 °C for 2 h. Afterwards, the feed gas was switched to 10% H_2_ in Ar. Subsequently, TPR was performed by raising the temperature from 50 to 800 °C at a rate of 10 °C min^−1^.

Electron paramagnetic resonance (EPR) spectroscopy was used to characterize EPR-active Cu^2+^ species in the f Cu-SSZ-13 catalysts. The EPR spectra were recorded on a Bruker EMXplus-6/1 spectrometer. Powder samples were directly loaded into quartz tubes and placed at the center of the resonator cavity. The spectra were collected in magnetic-field sweep mode with a sweep width of 6000 G.

*In situ* diffuse reflectance infrared Fourier transform spectroscopy (*in situ* DRIFTS) was carried out on a Nicolet iS50 FTIR spectrometer (Thermo Fisher Scientific) equipped with an *in situ* diffuse reflectance reaction cell to monitor surface adsorbed species and transient reaction processes over the Cu-SSZ-13 catalysts. Approximately 30 mg of catalyst powder was loaded into the sample cup and pretreated at 550 °C under N_2_ flow (200 mL min^−1^) for 1 h to remove surface impurities and adsorbed water. After the sample was adjusted to the target temperature, a background spectrum was collected under N_2_. The spectra were recorded in the range of 4000–600 cm^−1^ with a resolution of 4 cm^−1^, and 64 scans were accumulated for each spectrum. Two transient experiments were performed. In the NH_3_ adsorption-reaction experiment, NH_3_ was first introduced until adsorption saturation, followed by N_2_ purging, and then NO + O_2_ was introduced to examine the reaction of pre-adsorbed NH_3_ species. In the NO + O_2_ co-adsorption–reaction experiment, NO + O_2_ was first introduced until adsorption saturation, followed by N_2_ purging, and then NH_3_ was introduced to evaluate the reactivity of pre-adsorbed NO_*x*_ species. The total flow rate was maintained at 200 mL min^−1^, and the feed gas contained 500 ppm NO, 550 ppm NH_3_, 10 vol% O_2_, and N_2_ as the balance gas when applicable.

### 2.4 NH_3_-SCR activity of prepared Cu-SSZ-13 zeolites

The evaluation of NH_3_-SCR activity was performed using a custom-built multifunctional synthetic gas bench. 0.5 g of each prepared sample (40–60 mesh) was loaded into a fixed-bed quartz reactor and then pretreated under N_2_ flow at 550 °C for 1 h with a space velocity of 240 000 h^−1^. After the furnace inlet temperature was cooled to 100 °C and held for 30 min, the activity test was initiated by switching the feed gas to the reaction gas (500 ppm NO, 550 ppm NH_3_, 10 vol% O_2_, 8 vol% CO_2_, 7 vol% H_2_O, and N_2_ as balance) and then heating the catalyst from 100 to 550 °C at a heating rate of 30 °C min^−1^. The outlet concentrations were measured at intervals of 50 °C using an online FTIR gas analyzer. Once the outlet NO concentration stabilized, the inlet temperature was adjusted to the next setpoint. The NO_*x*_, NH_3_ conversions and N_2_ selectively were calculated as follows:7
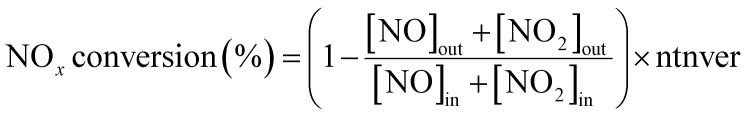
8
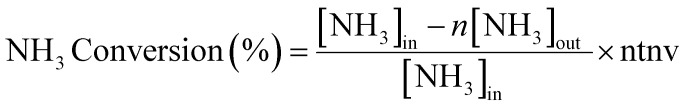

9 NO_2_ concentration = *C*(NO_2-out_ − NO_2-in_)

where *C* is the concentration of a given component, and the subscripts in and out denote the inlet and outlet positions, respectively.

## 3 Results and discussion

### 3.1 Characterization

#### 3.1.1 Surface area and pore structure of prepared Cu-SSZ-13 zeolites

To investigate the influence of the Cu/Al ratio on the textural properties of Cu-SSZ-13 zeolite and its stability during hydrothermal aging, N_2_ physisorption and ICP-OES measurements were performed on the prepared samples. The results are summarized in Table 1. As the Cu/Al ratio increased, both the BET specific surface area and the micropore volume of the samples gradually decreased. When the Cu/Al ratio was raised from 0.3 to 0.4, the BET specific surface area of the fresh samples decreased from 593 m^2^ g^−1^ to 572 m^2^ g^−1^, and the pore volume decreased from 0.31 cm^3^ g^−1^ to 0.28 cm^3^ g^−1^. This is likely because the introduced copper species partially occupied the cation exchange sites within the CHA cages and channels, while a small amount of agglomerated copper oxides may have attached to the pore mouths or external surfaces, causing physical blockage of micropores.^30^ The pore size distribution of all fresh samples was centered around 0.38 nm, which is consistent with the characteristic micropore size of SSZ-13 zeolite, indicating that under an appropriate Cu/Al ratio (≤0.3), the introduction of copper did not alter the microporous structure of the CHA framework. After the catalysts were subjected to hydrothermal aging at 800 °C for 16 h, the BET specific surface area and micropore volume of the aged samples decreased by approximately 5% compared to the fresh samples. The Cu–Al-4 sample showed a more significant decrease in specific surface area after aging, likely because the excessive copper species migrated and aggregated under hydrothermal conditions to form large CuO particles, which blocked the micropores and accelerated structural degradation.

#### 3.1.2 Phase structure of prepared Cu-SSZ-13 zeolites

**Fig. 1:**
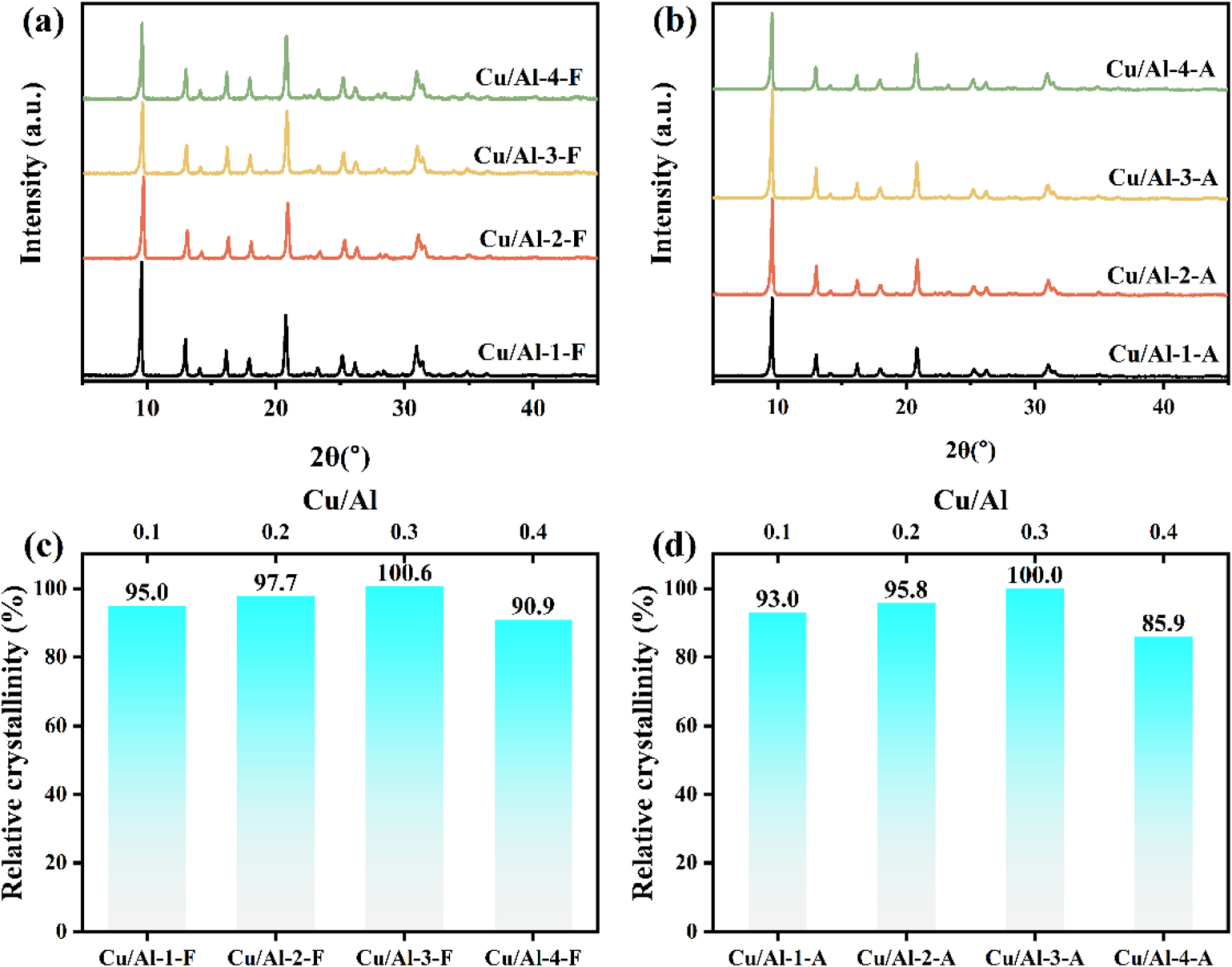
XRD patterns of (a) XRD patterns of fresh samples with different Cu/Al ratios; (b) XRD patterns of aged samples with different Cu/Al ratios; (c) comparison of fresh catalyst crystallinity; (d) comparison of aged catalyst crystallinity.

The XRD patterns of fresh and hydrothermally aged Cu-SSZ-13 with different Cu/Al ratios are shown in Fig. 1. All samples exhibited characteristic diffraction peaks of the CHA topology at 2*θ* = 9.5°, 12.9°, 16.2°, 25.1°, and 30.98°, corresponding to the (100), (101), (104), (021), (211), (131), and (401) crystal planes, respectively.^31–33^ No characteristic peaks of CuO or Cu_2_O were detected in the patterns, likely because the copper species existed as highly dispersed ion-exchanged species or subnanometer clusters without forming a distinct crystalline phase, or because the particle size was below the detection limit of XRD.^34^ After hydrothermal aging at 800 °C for 16 h, the intensities of the characteristic CHA peaks of the samples decreased to varying degrees, but the peak positions remained intact. The relative crystallinity of the aged samples decreased significantly compared to the fresh samples, and the extent of the decrease was closely related to the Cu/Al ratio. When Cu/Al ≤ 0.3, the relative crystallinity of the aged samples decreased by approximately 5%, demonstrating excellent hydrothermal stability. Previous studies have shown that at low Cu/Al ratios, an appropriate amount of Cu^2+^ ions occupies the 6MR and 8MR sites, enhancing framework stability by balancing the negative charge generated by dealumination and inhibiting hydrolysis reactions.^35^ When Cu/Al > 0.3, the relative crystallinity of the aged catalysts decreased by about 10%, with a particularly severe loss of intensity at the low-angle diffraction peak around 9.5° associated with micropores. This indicates that excess copper species likely migrated and locally aggregated during hydrothermal aging, promoting framework hydrolysis and pore blockage, thereby accelerating the loss of crystallinity.

#### 3.1.3 Morphology of the prepared Cu-SSZ-13 zeolites

**Fig. 2:**
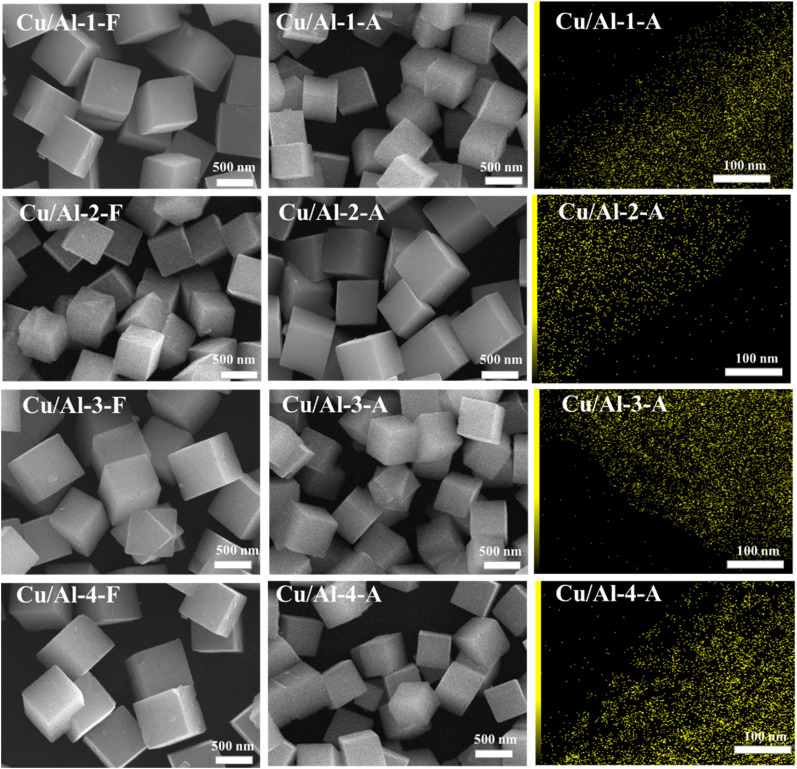
SEM images of fresh and aged Cu/Al samples, together with corresponding Cu elemental mapping.

The morphology and crystallite size of fresh and aged Cu-SSZ-13 catalysts with different Cu/Al ratios were analyzed by SEM and HRTEM. From the SEM images shown in Fig. 2, all fresh catalyst samples exhibited regular cubic morphology with smooth crystal surfaces and good inter-crystal dispersion, and no obvious agglomeration or amorphous phases were observed. Statistical analysis of crystallite sizes revealed that all samples had a uniform crystallite size distribution with an average diameter of approximately 0.25 µm, and no significant differences were found among samples with different Cu/Al ratios, indicating that gradient Cu loading did not affect the initial crystal morphology or size of the CHA-type zeolite. To further investigate the changes in morphology and crystallite size after hydrothermal aging, all catalyst samples were tested after aging at 800 °C for 16 h. The SEM images show that, except for the Cu/Al-4 sample, the hydrothermally aged samples still remained intact cubic crystal morphology with clear crystal outlines, and no obvious signs of degradation such as crystal fragmentation, edge rounding, or grain boundary fusion were observed. Statistical results indicated that the average crystallite size of the aged samples did not increase significantly compared to the fresh samples, and no large-area densified regions or disordered agglomerates caused by framework collapse were observed. These results demonstrate that the prepared Cu-SSZ-13 catalyst exhibits excellent morphology retention ability at the micro-to sub-micron scale, indicating good hydrothermal stability.^36^

**Fig. 3:**
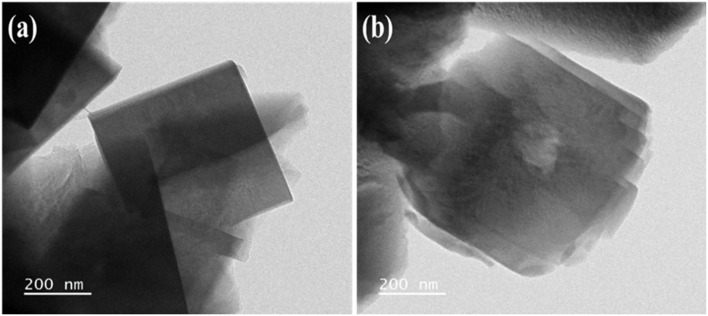
(a) TEM image of fresh Cu/A-4 sample; (b) TEM image of HTA Cu/A-4 sample.

Based on the SEM results, to further investigate the effect of the Cu/Al ratio on catalyst morphology, a comparative HRTEM analysis was performed on the fresh and hydrothermally aged Cu/Al-4 sample, as shown in Fig. 3. The fresh sample exhibits a typical cubic morphology with clear crystal contours and a dense, uniform internal structure, free of obvious defects or amorphous phases, indicating an intact zeolite framework structure. After hydrothermal aging at 800 °C for 16 h, as seen in Fig. 3(b), the crystal edges and corners are significantly rounded, the crystal facet edges become rough, and step-like defects and local erosion appear on the originally cubic crystal facets. These observations suggest that dealumination and local framework collapse occurred during hydrothermal aging, leading to a decrease in structural order. Furthermore, no micron-sized CuO agglomerates are observed; however, the changes in morphology and contrast indicate irreversible damage to the crystal structure of Cu-SSZ-13 caused by hydrothermal aging, which may consequently affect its catalytic activity and stability.^23,37,38^

#### 3.1.4 The NH_3_-TPD profiles of the prepared Cu-SSZ-13 zeolites

**Fig. 4:**
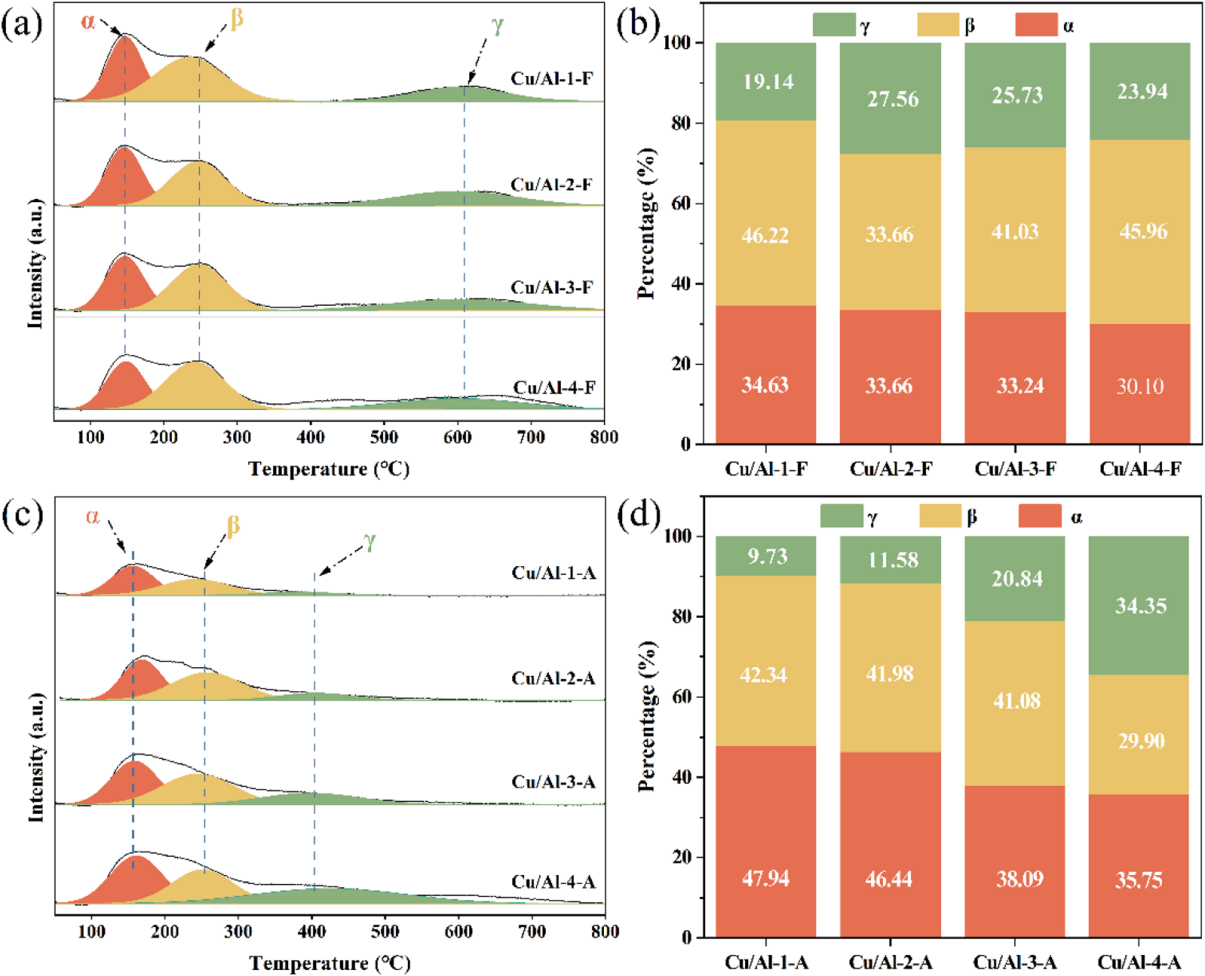
(a) NH_3_-TPD results of the fresh samples; (b) the relative percentage of the fresh samples; (c) NH_3_-TPD results of the aged samples; (d) the relative percentage of the aged samples.

The NH_3_-TPD results for fresh and aged samples with different Cu/Al ratios are shown in Fig. 4. Both fresh and aged catalysts exhibit three typical NH_3_ desorption peaks. Peaks α below 200 °C, attributed to physisorbed NH_3_ and NH_3_ species weakly interacting with copper species; peaks β between 200 and 350 °C, assigned to NH_3_ adsorbed on cationic sites of the zeolite; and peaks γ between 400 and 550 °C, mainly attributed to strongly bound NH_3_ on Brønsted acid sites (framework Si–OH–Al).^38–40^

For the fresh catalysts, as the Cu/Al ratio increases from 0.1 to 0.4, the relative proportion of the α desorption peak decreases from 34.63% to 30.10%, the proportion of the β desorption peak first decreases and then increases, while the proportion of the γ desorption peak first increases and then decreases, reaching a maximum of 27.56% at Cu/Al = 0.2. This trend is consistent with literature reported.^41,42^ When the Cu/Al ratio is controlled within 0.2–0.3, copper species remain well dispersed in the 8MR cages of the zeolite, and the number of isolated Cu^2+^ sites is sufficient.^43^ This reduces the formation of weakly interacting copper species while preserving the framework Brønsted acid sites to the greatest extent, allowing different types of adsorption sites to achieve effective synergistic effects.^44^ After hydrothermal aging at 800 °C for 16 h, the distribution of NH_3_ adsorption sites in all samples undergoes significant reconstruction. For the aged samples, the proportion of γ desorption peak increases markedly with increasing Cu/Al ratio, rising from 9.73% for the Cu/Al = 0.1 sample to 34.35% for the Cu/Al = 0.4 sample. The proportion of low-temperature peak α is overall higher than that of the fresh samples and decreases from 47.94% to 35.75% as the Cu/Al ratio increases. The proportion of medium-temperature peak β is generally lower than that of the fresh samples and continuously decreases from 42.34% to 29.90% with increasing Cu/Al ratio. For samples with low Cu/Al ratios (0.1–0.2), the proportion of high-temperature peak γ declines sharply after aging, indicating a loss of Brønsted acid sites, while migration and aggregation of copper species occur, leading to a significant increase in the proportion of weakly interacting adsorption sites. The Cu/Al = 0.3 sample shows an adsorption site structure after aging that is close to that of the fresh sample. Based on the combined characterization results of fresh and aged samples, the Cu-SSZ-13 catalyst with Cu/Al = 0.3 maintains abundant active sites such as Brønsted acid sites and isolated Cu^2+^ species, even under severe hydrothermal conditions, demonstrating excellent hydrothermal stability.

#### 3.1.5 The H_2_-TPR profiles of the prepared Cu-SSZ-13 zeolites

**Fig. 5:**
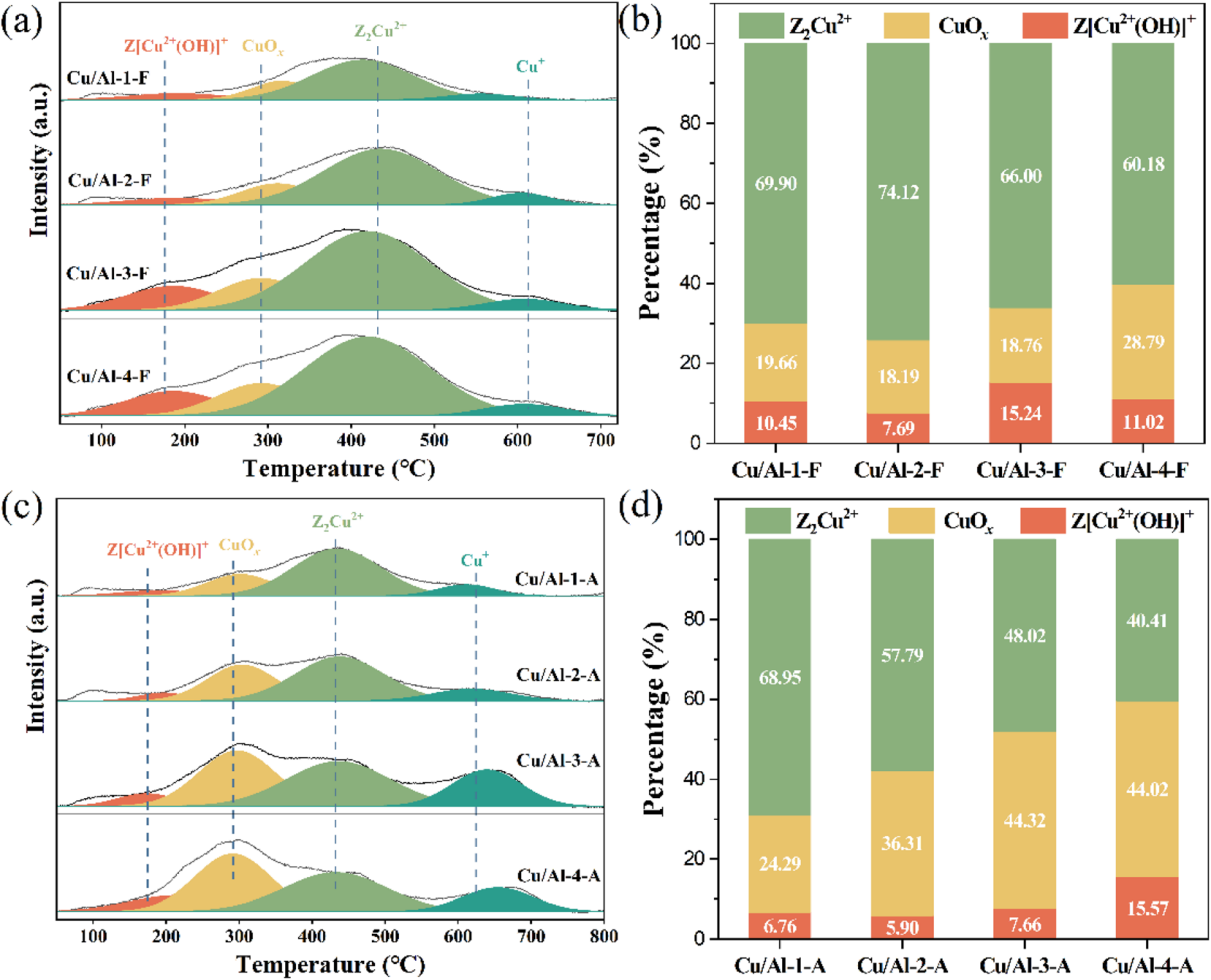
(a) H_2_-TPR results of the fresh samples; (b) the relative percentage of the fresh samples; (c) H_2_-TPR results of the fresh samples; (d) the relative percentage of the fresh samples.

The H_2_-TPR results for fresh and hydrothermally aged Cu-SSZ-13 catalysts with different Cu/Al ratios are shown in Fig. 5. Previous studies^45^ have shown that the H_2_-TPR profiles of Cu-SSZ-13 can be divided into several characteristic reduction regions. Specifically, the reduction peak below 200 °C is attributed to the reduction of Z[Cu^2+^(OH)]^+^ active species inside the zeolite cages (Cu^2+^ → Cu^+^). The medium-temperature peak at 250–350 °C corresponds to the reduction of highly dispersed CuO_*x*_ clusters on the zeolite surface and in the pores (Cu^2+^ → Cu^0^). The high-temperature peak at 350–550 °C is the characteristic reduction signal of the stable Z_2_Cu^2+^ species located in the 6MR (Cu^2+^ → Cu^+^). The reduction peak above 550 °C is assigned to the deep reduction of Cu^2+^ → Cu^+^, or to the reduction of inert CuAlO_*x*_ composite oxides formed by the interaction between copper and framework aluminum during hydrothermal aging.^35,45^ The intensity of this signal can be used as a comprehensive indicator of the relative content of hardly reducible agglomerated copper species and Cu–Al inert complexes.

For the fresh catalysts, as the Cu/Al ratio increases from 0.1 to 0.4, the proportion of Z_2_Cu^2+^ active species first increases and then decreases, reaching a maximum at Cu/Al = 0.2. The proportion of CuO_*x*_ clusters increases continuously with increasing copper loading, while the proportion of Z[Cu^2+^(OH)]^+^ species remains low throughout. Moreover, no significant reduction signal above 550 °C is observed for fresh sample, indicating that the copper species in the fresh state are predominantly highly dispersed ion-exchanged Cu^2+^. After hydrothermal aging at 800 °C for 16 h, a significant restructuring of copper species occurs. For samples with low Cu/Al ratios (0.1–0.2), the highly stable Z_2_Cu^2+^ species remain dominant, the increase in CuO_*x*_ clusters is minor, and the intensity of the reduction peak above 550 °C is weak, indicating that only a small amount of hardly reducible agglomerated copper species is formed.^46^ Thus, the overall structural stability of copper species is well preserved. In contrast, for samples with Cu/Al ratio of 0.4, a substantial loss of Z_2_Cu^2+^ active sites occurs, the proportion of CuO_*x*_ agglomerates increases significantly, and the reduction peak above 550 °C is markedly enhanced, with its intensity gradually increasing with the Cu/Al ratio. This suggests that excess copper species not only undergo migration, aggregation, and sintering under high-temperature hydrothermal conditions, converting active Cu^2+^ species into Cu^+^ and aggregated copper species with no SCR activity, but also strongly interact with dealuminated framework aluminum to form thermodynamically stable CuAlO_*x*_ composite oxides, further causing irreversible deactivation of active sites.^47^

In summary, a low Cu/Al ratio in Cu-SSZ-13 effectively suppresses the aggregation of copper species, their deep reduction, and the formation of Cu–Al inert complexes during hydrothermal treatment, thereby maintaining a sufficient number of active Cu^2+^ sites. Conversely, an excessively high Cu/Al ratio greatly accelerates the loss of active species and the generation of inert byproducts, significantly deteriorating the hydrothermal stability of the catalyst. These results are in good agreement with the preceding acidity characterization and the subsequent deNO_*x*_ performance data.

#### 3.1.6 The UV-vis DRS of the prepared Cu-SSZ-13 zeolites

**Fig. 6:**
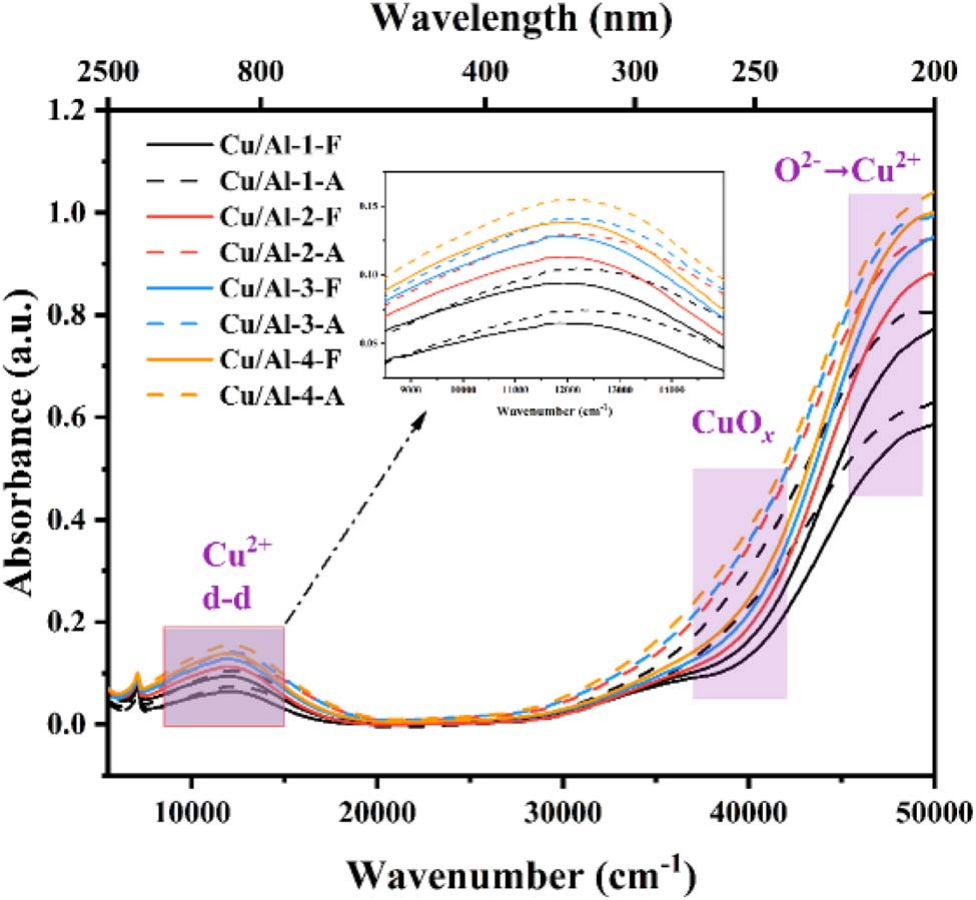
(a) UV-vis DRS spectra of the prepared samples.

Fig. 6 shows the UV-vis DRS spectra of fresh and aged Cu-SSZ-13 samples with different Cu/Al ratios. All samples exhibit typical absorption features of copper species. Three distinct peaks are observed at approximately 12 500, 40 000, and 48 000 cm^−1^, which correspond to (i) the d–d electronic transition of isolated Cu^2+^ species; (ii) the O^2−^ → Cu^2+^ charge-transfer transition of oligomeric CuO_*x*_ species (including Cu–O–Cu or O–Cu–O); and (iii) the d–d transitions of Cu^2+^ ions in an octahedral environment (also involving Cu–O–Cu or O–Cu–O charge transitions), respectively, as reported in ref. 48.

As the Cu/Al ratio increases from 0.1 to 0.4, the absorption intensities in all three regions increase significantly, indicating that not only does the loading of copper species increase, but the proportions of oligomeric and bulk CuO species also rise, with a clear tendency toward copper aggregation especially in the samples with high Cu/Al ratios.^49^ Furthermore, the absorption intensities of the aged samples are generally higher than those of the fresh ones, and the increase is more pronounced in the medium- and high-wavenumber regions. This indicates that the hydrothermal aging process promotes the migration and aggregation of Cu^2+^, generating more oligomeric CuO_*x*_ and bulk CuO.^50^ The copper aggregation is more severe in the samples with high Cu/Al ratios. In the catalysts with low Cu/Al ratios, isolated Cu^2+^ species dominate, resulting in optimal copper dispersion and superior resistance to hydrothermal aging. In contrast, although the high Cu/Al ratio samples possess higher copper loadings, the excess copper species tend to aggregate during aging, which may adversely affect the high-temperature selectivity and stability of the catalysts.

#### 3.1.7 EPR spectra of the prepared Cu-SSZ-13 zeolites

**Fig. 7:**
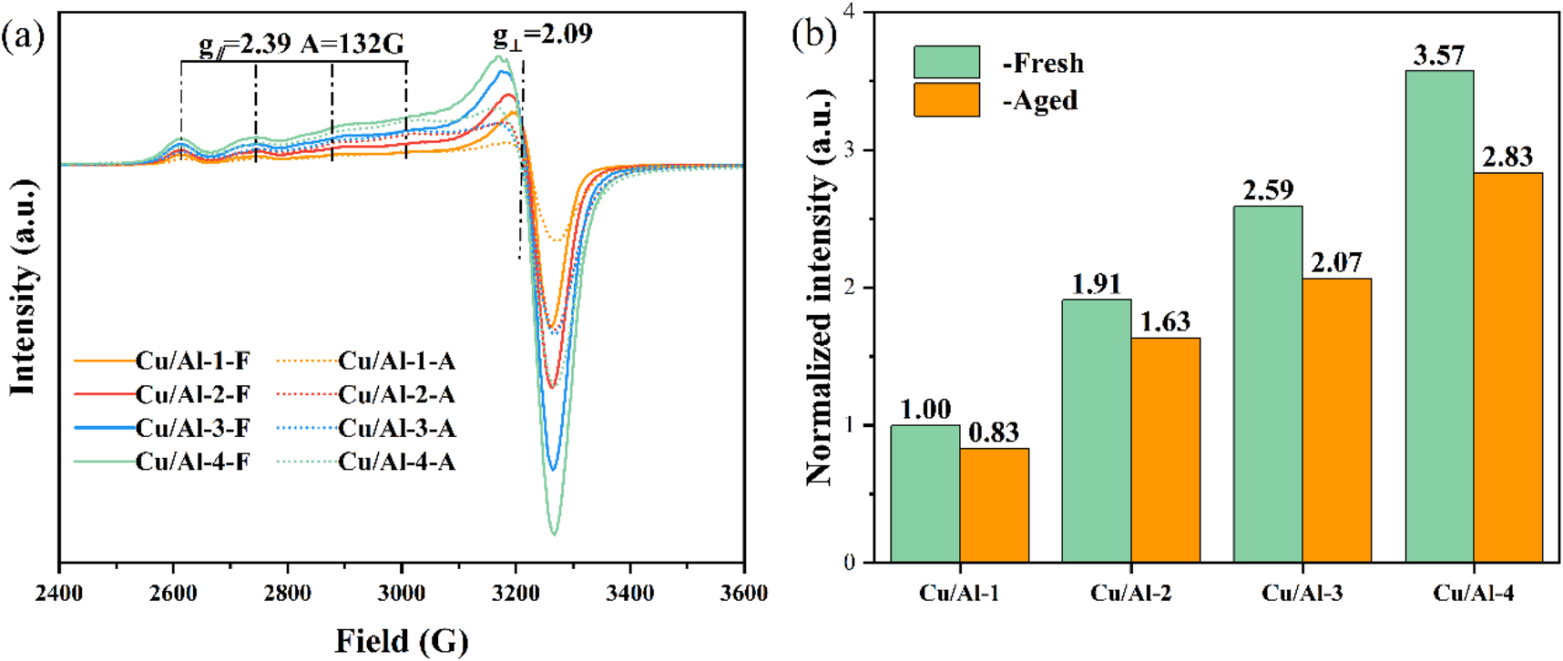
(a) EPR spectra of fresh and hydrothermally aged Cu-SSZ-13 catalysts with different Cu/Al ratios. (b) Normalized EPR intensity derived from double integration of the EPR spectra (I/I_Cu/Al-1-F_).

Although UV-vis DRS provides useful information on isolated Cu^2+^ and CuO_*x*_-like species, the broad absorption bands make it difficult to evaluate the evolution of Cu^2+^ species unambiguously.^48^ Therefore, EPR spectroscopy was further employed to probe EPR-active Cu^2+^ species in the fresh and aged catalysts. EPR spectroscopy was further employed to probe the evolution of EPR-active Cu^2+^ species in the fresh and hydrothermally aged Cu-SSZ-13 catalysts.^26,51^ As shown in Fig. 7(a), all samples exhibited characteristic Cu^2+^ signals, indicating that highly dispersed Cu^2+^ species were present in the Cu-SSZ-13 catalysts after Cu ion exchange and could still be detected after hydrothermal aging.

To compare the relative variation of EPR-active Cu^2+^ species, the baseline-corrected derivative EPR spectra were double-integrated and normalized to that of Cu/Al-1-F, which was set as 1.000 (Fig. 7(b)).^45,51^ For the fresh catalysts, the normalized double-integrated EPR areas increased from 1.000 to 1.911, 2.589, and 3.569 with increasing Cu/Al ratio from Cu/Al-1-F to Cu/Al-4-F, respectively. This trend indicates that increasing Cu loading generally increases the amount of EPR-detectable Cu^2+^ species. However, the stronger EPR signal at high Cu/Al ratio should not be directly interpreted as a proportional increase in selective SCR-active Cu sites. Combined with the UV-vis DRS and H_2_-TPR results, the high-Cu samples also contain more CuO_*x*_-like or less selectively reducible Cu species, which may promote non-selective NH_3_ oxidation and contribute to N_2_O formation.^20,21^

After hydrothermal aging, the normalized EPR areas of Cu/Al-1-A, Cu/Al-2-A, Cu/Al-3-A, and Cu/Al-4-A were 0.837, 1.634, 2.072, and 2.834, respectively. Although the aged samples still followed an overall increasing trend with Cu/Al ratio, their EPR intensities decreased compared with the corresponding fresh samples. The EPR signal retentions were approximately 83.7%, 85.5%, 80.0%, and 79.4% for Cu/Al-1, Cu/Al-2, Cu/Al-3, and Cu/Al-4, respectively. This result suggests that hydrothermal aging induces redistribution, migration, or coordination reconstruction of EPR-active Cu^2+^ species.^24,52^ The relatively higher retention of Cu/Al-2 indicates that an intermediate Cu/Al ratio helps maintain a more stable Cu^2+^ environment after aging, whereas excessive Cu loading makes Cu species more susceptible to hydrothermal reconstruction and aggregation.^52^

These EPR results provide additional evidence that N_2_O suppression over Cu-SSZ-13 is not achieved by simply maximizing the amount of EPR-active Cu^2+^ species, but by maintaining an appropriate and hydrothermally stable Cu^2+^ environment that selectively participates in the SCR cycle.

#### 3.1.8 The XPS profiles of the prepared Cu-SSZ-13 zeolites

**Fig. 8:**
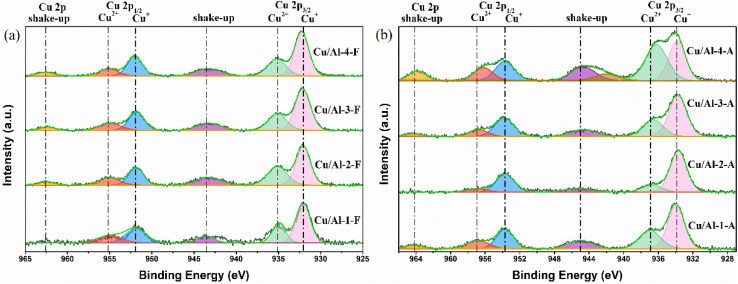
(a) XPS spectra of the fresh samples; (b) XPS spectra of the aged samples.

Qualitative evaluation of the Cu species in fresh and hydrothermally aged Cu-SSZ-13 samples with varying Cu/Al ratios was conducted *via* XPS, with the Cu 2p spectra presented in Fig. 8(a and b). As shown in Fig. 8(a), satellite peak characteristic of Cu^2+^ were observed at approximately 943 eV, situated approximately 10 eV above the respective Cu 2p_3_/_2_ main peaks, in agreement with ref. 49. These shake-up satellite arise from charge transfer between the metal 3d orbitals and the surrounding ligand O 2p orbitals. The main Cu 2p_3_/_2_ and Cu 2p_1_/_2_ peaks, located at approximately 933.7 ± 0.2 eV and 953.6 ± 0.2 eV, are characteristic of Cu^2+^ species, consistent with previous reports.^49,50,53^ The Cu 2p_3_/_2_ spectra were further deconvoluted into two components: the lower-binding-energy peak at approximately 932.4 eV is attributed to Cu^+^ species, whereas the higher-binding-energy peak at approximately 933.6 eV corresponds to isolated Cu^2+^.^50^ Based on the peak fitting results of the Cu 2p_3_/_2_ spectra, the isolated Cu^2+^ and Cu^+^ were quantified by integrating their respective peak areas and calculating their relative contributions, with the results summarized in Table 2. The Cu 2p_3_/_2_ XPS peak fitting results indicate that for fresh Cu-SSZ-13 samples, as the Cu/Al ratio increases from 0.1 to 0.4, the relative content of Cu^+^ continuously decreases from 74.61% to 43.85%, while the proportion of Cu^2+^ gradually rises from 25.39% to 56.15%, and the Cu^+^/Cu^2+^ ratio drops from 2.94 to 0.78. This suggests that at low Cu/Al ratios, Cu species primarily anchor in the acidic sites of the support as Cu^+^. When the loading exceeds the active site capacity, Cu species aggregate and form Cu^2+^ and bulk CuO. After hydrothermal aging at 800 °C for 16 h, all samples exhibit a trend of Cu^+^ consumption and an overall increase in Cu^2+^ content, with the Cu^2+^/Cu^+^ ratio significantly lower than that of the fresh state. Notably, the high-load sample Cu/Al-4-A shows a Cu^2+^ proportion as high as 73.35%, and the prominent CuO characteristic peak at 933.6 eV in the spectra confirms that high-temperature aging promotes the conversion of Cu^+^ to Cu^2+^ and the migration-aggregation of Cu species, forming large CuO crystallites. Overall, the sample of Cu/Al-2 maintain a superior Cu^+^/Cu^2+^ ratio and better hydrothermal aging resistance and NH_3_-SCR performance.

**Table 2:** Cu 2p_3/2_ XPS peak fitting results

Sample	Percentage%		Cu^+^/Cu^2+^
	Cu^+^	Cu^2+^	
Cu/Al-1-F	74.61	25.39	2.94
Cu/Al-2-F	65.24	34.76	1.88
Cu/Al-3-F	53.87	46.13	1.17
Cu/Al-4-F	43.85	56.15	0.78
Cu/Al-1-A	60.21	39.79	1.51
Cu/Al-2-A	48.53	51.47	0.94
Cu/Al-3-A	34.61	65.39	0.53
Cu/Al-4-A	26.65	73.35	0.36

### 3.2 The effect of Cu/Al ratios in NH_3_-SCR

**Fig. 9:**
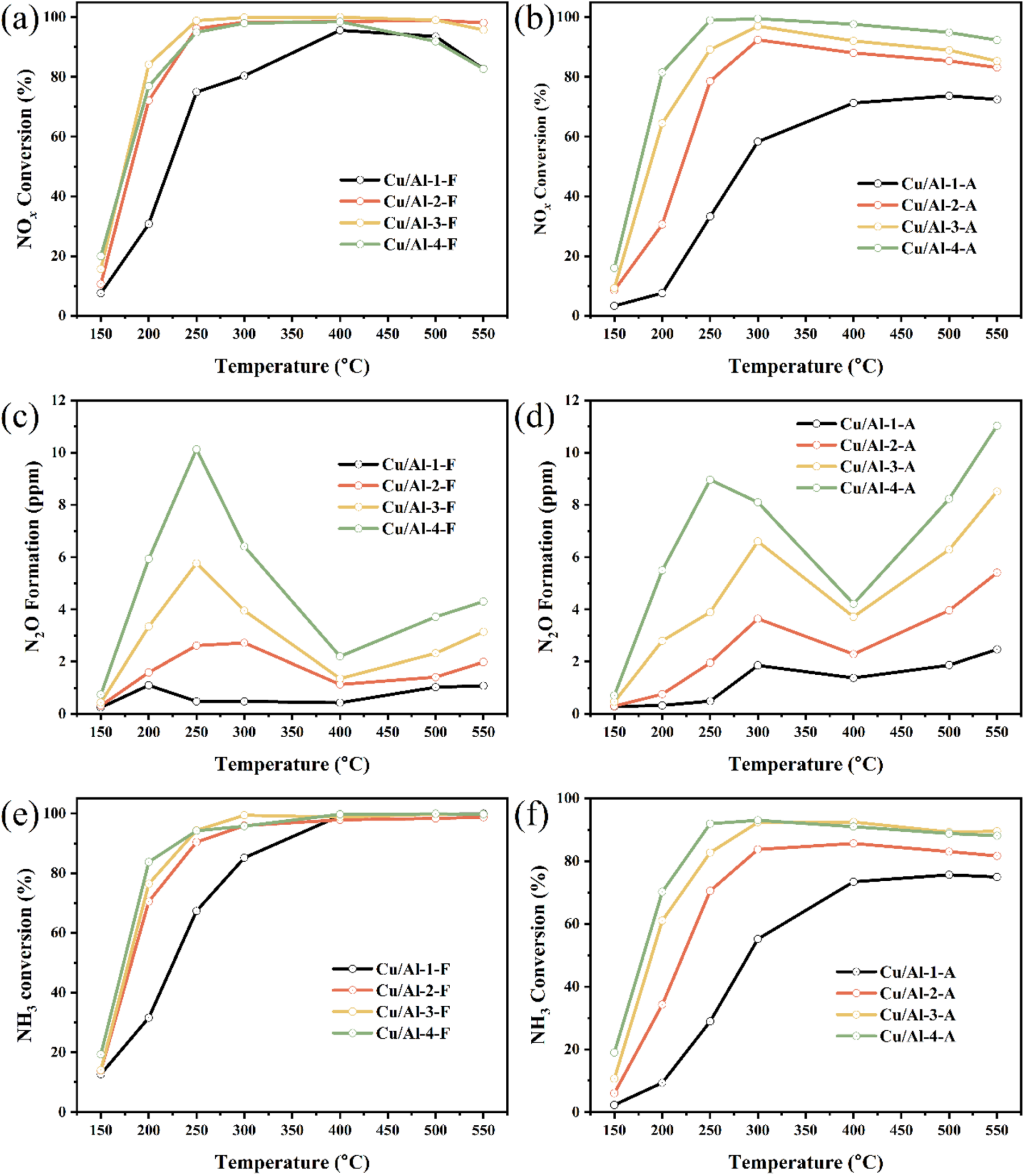
(a and b) The NO_*x*_ conversion of fresh and aged samples; (c and d) the N_2_O formation of fresh and aged samples; (e and f) the NH_3_ conversion of fresh and aged samples.

To further elucidate the influence of the Cu/Al ratio on N_2_O formation over Cu-SSZ-13 catalysts and to provide theoretical supports for the development of NH_3_-SCR catalysts with efficient N_2_O suppression, a systematic comparison was conducted on the fresh and aged performance of Cu-SSZ-13 catalysts with SAR = 21 and different Cu/Al ratios. The results are shown in Fig. 9. As the Cu/Al ratio increased from 0.1 to 0.3, both the fresh and hydrothermally aged catalysts exhibited a consistent trend in NO_*x*_ and NH_3_ conversion efficiency, *i.e.*, a significant improvement in the LT range. When the Cu/Al ratio was further increased to 0.4, the NO_*x*_ and NH_3_ conversion efficiency in the HT region decreased markedly, indicating that excessive Cu loading is detrimental to the high-temperature catalytic activity of the catalyst.^53^

The variation of N_2_O formation concentration with Cu/Al ratio for fresh and aged catalysts is shown in Fig. 9(e and f). The results indicate a significant positive correlation between N_2_O formation and the Cu/Al ratio for both fresh and aged catalysts. For the fresh catalysts, the Cu/Al = 0.1 sample exhibited extremely low N_2_O formation across the entire temperature range. As the Cu/Al ratio increased, the peak N_2_O formation increased significantly, while the decrease in N_2_O formation after 400 °C was associated with enhanced thermal decomposition of N_2_O at high temperatures. After hydrothermal aging at 800 °C for 16 h, N_2_O formation increased significantly for all samples, and the effect of Cu loading was further amplified. The Cu/Al-4-A sample showed N_2_O formation close to 11 ppm at 550 °C, whereas Cu/Al-1-A remained below 2.5 ppm. This phenomenon is mainly attributed to the dealumination of the zeolite framework during hydrothermal aging, which causes the migration and agglomeration of the ion-exchanged isolated Cu^2+^ ions into large CuO_*x*_ particles or CuAlO_*x*_ composite oxides. These species exhibit significantly higher NH_3_ oxidation activity than isolated Cu^2+^ ions and are more prone to catalyze N_2_O formation through non-selective oxidation pathways, leading to a sharp increase in N_2_O emissions after aging for samples with high Cu/Al ratios.

### 3.3 *In situ* DRIFTS analysis of surface intermediate reactivity

At high temperatures, N_2_O formation over Cu-SSZ-13 is mainly related to non-selective NH_3_ oxidation promoted by strongly oxidative Cu species.^54^ In contrast, low-temperature N_2_O formation is closely associated with the accumulation, transformation, and decomposition of surface NH_3_/NO_*x*_-derived intermediates, such as nitrate/nitrite- and NH_4_NO_3_-related species.^21,28^ Therefore, to further clarify the relationship between Cu-species evolution and N_2_O formation, 200 °C was selected as a representative low-temperature condition for *in situ* DRIFTS measurements. NH_3_ adsorption, NO + O_2_ adsorption, and transient surface reactions between pre-adsorbed intermediates and reactant gases were investigated to reveal how Cu/Al regulation and hydrothermal aging affect the surface reaction balance between NH_3_ activation and NO_*x*_ reduction.

#### 3.3.1 Effect of Cu/Al ratio on NH_3_ adsorption and NO_*x*_ activation

**Fig. 10:**
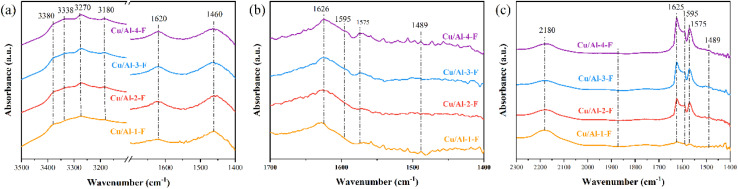
*In situ* DRIFTS spectra of fresh Cu/Al–F catalysts with different Cu/Al ratios at 200 °C: (a) NH_3_ adsorption, (b) reaction between pre-adsorbed NH_3_ species and NO + O_2_, and (c) NO + O_2_ adsorption.

*In situ* DRIFTS was first employed to investigate the influence of Cu/Al ratio on NH_3_ adsorption and NO_*x*_ activation over the fresh Cu/Al–F catalysts at 200 °C. As shown in Fig. 10(a), after NH_3_ adsorption, all catalysts exhibited typical NH_3_-related bands. The bands at around 3380, 3338, 3270, and 3180 cm^−1^ can be assigned to the N–H stretching vibrations of adsorbed NH_3_/NH_4_^+^ species.^48,54^ In the fingerprint region, the band at around 1620 cm^−1^ is attributed to NH_3_ adsorbed on Lewis acid sites, while the band at around 1460 cm^−1^ corresponds to NH_4_^+^ species formed on Brønsted acid sites.^48,54^ With increasing Cu/Al ratio, the NH_3_ adsorption features changed obviously, indicating that Cu introduction modified the distribution of NH_3_ adsorption sites. In particular, the Lewis-acid-related NH_3_ band became more evident, suggesting that Cu-associated Lewis acid sites played an increasingly important role in NH_3_ adsorption and activation.^55^

After NH_3_ adsorption saturation followed by NO + O_2_ introduction, the NH_3_-related bands decreased and new bands associated with NO_*x*_-derived species appeared, as shown in Fig. 10(b). The band around 1625 cm^−1^ can be assigned to overlapping contributions from residual Lewis-bound NH_3_ and newly formed NO_*x*_-derived species. The bands at around 1595 and 1575 cm^−1^ are related to nitrate/nitrite species, while the band at around 1489 cm^−1^ can be associated with nitrate or NH_4_NO_3_-related intermediates. Compared with the low-Cu samples, the high-Cu samples, especially Cu/Al-4-F, showed more pronounced bands in the 1625–1575 and 1489 cm^−1^ regions after NO + O_2_ introduction. This indicates that increasing Cu/Al ratio enhances the formation or stabilization of NO_*x*_-derived intermediates during the reaction between pre-adsorbed NH_3_ species and incoming NO + O_2_.^23,56^

The NO + O_2_ adsorption spectra further confirmed the stronger NO_*x*_ activation ability of the high-Cu samples. As shown in Fig. 10(c), bands at around 1625, 1595, 1575, and 1489 cm^−1^ were observed after NO + O_2_ adsorption, which can be assigned to nitrate/nitrite-related surface species. In addition, the band at around 2180 cm^−1^ can be attributed to NO+ or nitrosyl-like species formed on Cu-related sites. The intensity of these NO_*x*_-related bands increased with increasing Cu/Al ratio, indicating that higher Cu loading promoted NO oxidation and the formation of surface NO_*x*_ intermediates.^21^

These results demonstrate that the Cu/Al ratio strongly affects both NH_3_ adsorption and NO_*x*_ activation over Cu-SSZ-13. An appropriate amount of Cu species is necessary for NH_3_ activation and NO_*x*_ reduction. However, excessive Cu loading enhances the formation of NO_*x*_-derived and NH_4_NO_3_-related intermediates. Since low-temperature N_2_O formation over Cu-SSZ-13 is closely associated with the formation and decomposition of NH_4_NO_3_-related species, the accumulation of these intermediates over high-Cu samples may increase the probability of non-selective pathways toward N_2_O formation.^28^ Therefore, the role of Cu species in N_2_O formation is not simply determined by the total amount of Cu, but by whether the Cu environment can maintain a balanced surface reaction between NH_3_ activation and NO_*x*_ reduction.

#### 3.3.2 Surface reaction behavior at 200 °C

**Fig. 11:**
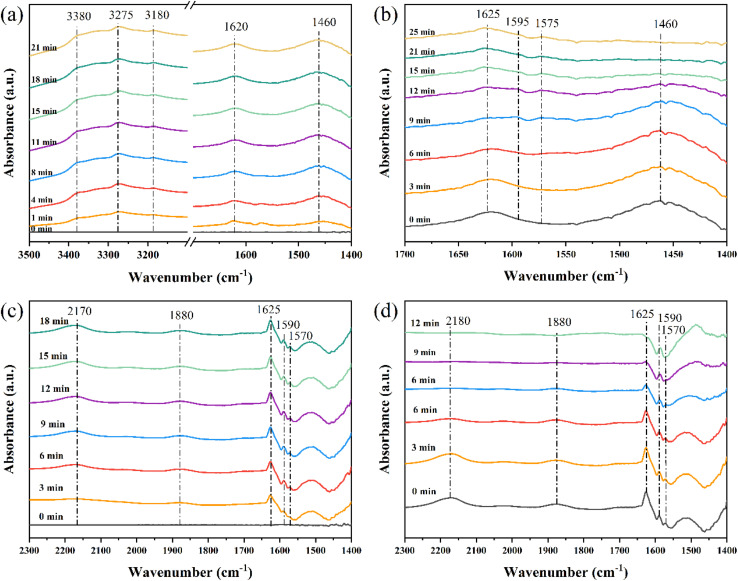
Time-resolved *in situ* DRIFTS spectra collected at 200 °C during transient surface reaction experiments: (a) NH_3_ adsorption, (b) reaction between pre-adsorbed NH_3_ species and NO + O_2_, (c) NO + O_2_ adsorption, and (d) reaction between pre-adsorbed NO_*x*_ species and NH_3_.

To further understand the low-temperature surface reaction behavior, time-resolved *in situ* DRIFTS experiments were conducted at 200 °C. As shown in Fig. 11(a), the NH_3_-related bands gradually increased with adsorption time, indicating that NH_3_ could be effectively adsorbed and stored on the catalyst surface at 200 °C. These adsorbed NH_3_/NH_4_^+^ species provide the surface reductants required for the subsequent SCR reaction.^55^

After NH_3_ adsorption saturation, NO + O_2_ was introduced to evaluate the reactivity of the pre-adsorbed NH_3_ species. As shown in Fig. 11(b), the NH_3_-related bands decreased gradually after switching to NO + O_2_, indicating that the pre-adsorbed NH_3_/NH_4_^+^ species participated in the reaction with incoming NO_*x*_. Meanwhile, NO_*x*_-derived bands became evident during the gas-switching process, suggesting that the reaction did not proceed only through direct consumption of adsorbed NH_3_, but also involved the formation and transformation of surface NO_*x*_-related intermediates. The coexistence of residual NH_3_ species and newly formed NO_*x*_-derived intermediates indicates that the low-temperature SCR process depends strongly on the balance between NH_3_ activation and NO_*x*_ activation.

NO + O_2_ adsorption was then performed to further clarify the formation behavior of NO_*x*_-derived species at 200 °C. As shown in Fig. 11(c), NO_*x*_-related bands gradually developed with adsorption time, confirming that NO could be activated in the presence of O_2_ to form surface nitrosyl, nitrate, or nitrite-related intermediates. These species are important participants in the low-temperature SCR reaction, but their excessive stabilization or accumulation may also increase the probability of side reactions.

After NO + O_2_ adsorption saturation, NH_3_ was introduced to examine the reactivity of the pre-adsorbed NO_*x*_ species. As shown in Fig. 11(d), the NO_*x*_-related bands changed obviously after NH_3_ introduction, demonstrating that the pre-adsorbed NO_*x*_ species could further react with NH_3_. However, part of these bands remained during the reaction, suggesting that some NO_*x*_-derived intermediates were relatively stable at 200 °C and could not be rapidly consumed by NH_3_.

Overall, the time-resolved DRIFTS results reveal that the surface reaction at 200 °C involves dynamic interaction between adsorbed NH_3_/NH_4_^+^ species and NO_*x*_-derived intermediates. The formation, transformation, and incomplete consumption of these intermediates indicate that low-temperature N_2_O formation is closely related to the surface reaction balance between NH_3_ activation and NO_*x*_ reduction. Therefore, maintaining rapid and balanced consumption of NH_3_/NO_*x*_-derived intermediates is essential for suppressing non-selective pathways toward N_2_O formation over Cu-SSZ-13.

#### 3.3.3 Effect of hydrothermal aging on NH_3_/NO_*x*_ intermediate evolution

**Fig. 12:**
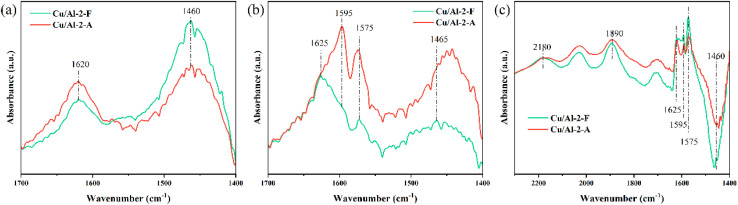
*In situ* DRIFTS comparison of fresh and hydrothermally aged Cu/Al-2 catalysts at 200 °C: (a) NH_3_ adsorption, (b) reaction between pre-adsorbed NH_3_ species and NO + O_2_, and (c) NO + O_2_ adsorption.

To further clarify the effect of hydrothermal aging on the surface reaction behavior related to N_2_O formation, the DRIFTS spectra of Cu/Al-2-F and Cu/Al-2-A were compared at 200 °C. As shown in Fig. 12(a), the NH_3_ adsorption features changed obviously after hydrothermal aging. Compared with Cu/Al-2-F, Cu/Al-2-A showed weaker NH_3_-related bands, especially in the Brønsted-NH_4_^+^ and Cu/Lewis-coordinated NH_3_ regions. This indicates that hydrothermal aging weakened the NH_3_ adsorption/storage ability and reconstructed the Cu-associated NH_3_ adsorption environment. Such changes are consistent with the loss of acid sites and the redistribution of Cu species induced by hydrothermal aging.^56^

After NH_3_ adsorption saturation followed by NO + O_2_ introduction, the difference between the fresh and aged samples became more pronounced, as shown in Fig. 12(b). For Cu/Al-2-F, the pre-adsorbed NH_3_ species were more readily consumed after exposure to NO + O_2_, indicating a relatively efficient reaction between adsorbed NH_3_ species and incoming NO_*x*_. In contrast, Cu/Al-2-A exhibited stronger residual NH_3_/NO_*x*_-derived features after NO + O_2_ introduction, particularly in the regions associated with nitrate/nitrite- and NH_4_NO_3_-related intermediates. This result suggests that hydrothermal aging weakens the rapid coupling between NH_3_ activation and NO_*x*_ reduction, leading to slower consumption or greater persistence of surface intermediates.

The NO + O_2_ adsorption spectra further confirm that hydrothermal aging changes the NO_*x*_ adsorption and activation behavior. As shown in Fig. 12(c), Cu/Al-2-A exhibited a different distribution of nitrosyl-like and nitrate/nitrite-related bands compared with Cu/Al-2-F. This indicates that the Cu sites responsible for NO_*x*_ adsorption and activation were reconstructed after hydrothermal aging. Rather than simply enhancing or weakening NO_*x*_ adsorption, aging changed the type, distribution, and stability of NO_*x*_-derived intermediates on the catalyst surface.

Combining the NH_3_ adsorption, NH_3_-to-NO + O_2_ switching, and NO + O_2_ adsorption results, it can be concluded that hydrothermal aging disturbs the surface reaction balance between NH_3_ activation and NO_*x*_ reduction. For the fresh catalyst, the adsorbed NH_3_ species and NO_*x*_-derived intermediates can be more effectively coupled through the SCR pathway. After aging, the reconstructed Cu environment leads to weaker NH_3_ storage, altered NO_*x*_ activation, and more persistent NH_3_/NO_*x*_-derived intermediates. These changes increase the probability of non-selective side reactions associated with low-temperature N_2_O formation.

Therefore, the DRIFTS results demonstrate that the increase in N_2_O formation after hydrothermal aging is not only related to the loss or migration of Cu species, but also to the aging-induced imbalance in surface intermediate evolution. Maintaining a stable Cu environment that enables rapid and balanced consumption of NH_3_/NO_*x*_-derived intermediates is essential for suppressing N_2_O formation over Cu-SSZ-13.^57^

### 3.4 The role of Cu species in N_2_O formation

The two isolated Cu^2+^ active species, Z[Cu^2+^(OH)]^+^ and Z_2_Cu^2+^, serve as the core active sites in Cu-SSZ-13 zeolite catalysts for NH_3_-SCR reactions. Both species exhibit excellent NO_*x*_ reduction capability with NH_3_ and low non-selective oxidation activity, effectively suppressing unwanted NH_3_ oxidation and N_2_O side reactions. However, the role of these ionic Cu species in N_2_O formation should not be evaluated only by their amount, but also by their ability to maintain a balanced surface reaction between NH_3_ activation and NO_*x*_ reduction. For fresh Cu-SSZ-13 catalysts, as the Cu/Al ratio increases from 0.1 to 0.4, the proportion of Z_2_Cu^2+^ initially rises and then decreases, peaking at Cu/Al = 0.2, while Z[Cu^2+^(OH)]^+^ remains consistently at low levels. When Cu/Al exceeds 0.3, the acid sites available for cation exchange in the zeolite become saturated, leaving excess Cu unable to participate in ion exchange, leading instead to gradual aggregation into CuO_*x*_ clusters. These CuO_*x*_ clusters possess poor SCR catalytic performance and strong non-selective NH_3_ oxidation activity, readily inducing N_2_O formation *via* side reactions. EPR confirms that the Cu/Al ratio and hydrothermal aging affect the distribution and stability of EPR-active Cu^2+^ species. However, a stronger EPR signal does not necessarily correspond to better N_2_O suppression, indicating that the selectivity of Cu species is more important than simply increasing the amount of Cu^2+^ species. *In situ* DRIFTS further shows that high-Cu samples exhibit more pronounced NO_*x*_-derived and nitrate/NH_4_NO_3_-related intermediates during NO + O_2_ adsorption and NH_3_-to-NO + O_2_ switching. Since low-temperature N_2_O formation over Cu-SSZ-13 is closely associated with the formation and decomposition of NH_4_NO_3_-related intermediates, excessive Cu loading may promote N_2_O formation by enhancing NO_*x*_ oxidation and disturbing the balance between NH_3_ activation and NO_*x*_ reduction. After hydrothermal aging at 800 °C for 16 h, the zeolite framework undergoes dealumination, breaking the coordination bonds between Cu^2+^ and framework Al atoms. Consequently, some Z[Cu^2+^(OH)]^+^ and Z_2_Cu^2+^ species leave the lattice, migrate, aggregate, and transform into CuO_*x*_. For high Cu/Al catalysts (above 0.3), excessive Cu further interacts with dealuminated framework Al to form thermodynamically stable, inert CuAlO_*x*_ composite oxides. This phase lacks SCR activity, is difficult to reduce, and exhibits strong non-selective ammonia oxidation ability, making it a key factor behind the sharp rise in N_2_O emissions after aging. DRIFTS after hydrothermal aging shows that aging weakens the efficient coupling between adsorbed NH_3_ species and incoming NO_*x*_. Combined with the EPR, H_2_-TPR, and UV-vis DRS results, this behavior can be attributed to the redistribution of ionic Cu species and the possible formation of CuO_*x*_-like or strongly interacting Cu species. These reconstructed Cu environments may promote non-selective NH_3_ oxidation and the persistence of nitrate/NH_4_NO_3_-related intermediates, thereby increasing N_2_O formation. An optimal Cu/Al ratio (preferably 0.2) maximizes retention of Z[Cu^2+^(OH)]^+^ and Z_2_Cu^2+^, inhibiting their conversion into CuO_*x*_ and CuAlO_*x*_, thereby achieving superior denitrification performance along with minimal N_2_O and NO_2_ byproduct emissions. Under high Cu/Al conditions, the transformation of the four active phases into inert phases intensifies, resulting in loss of active ionic species and accumulation of CuO_*x*_ and CuAlO_*x*_, directly causing degradation of high-temperature SCR activity and massive N_2_O generation.

## 4 Conclusions

In this work, Cu-SSZ-13 catalysts with different Cu/Al ratios were systematically investigated before and after hydrothermal aging to clarify the relationship between Cu-species evolution, NH_3_-SCR performance, and N_2_O formation. The catalytic results demonstrate that the Cu/Al ratio plays a decisive role in balancing NO_*x*_ reduction activity and N_2_O suppression. Insufficient Cu loading limits the number of redox-active Cu sites required for low-temperature SCR, whereas excessive Cu loading promotes the formation of CuO_*x*_-like species and enhances non-selective reaction pathways, leading to increased N_2_O formation. Among the investigated catalysts, Cu/Al-2 exhibited the most favorable balance between SCR activity, hydrothermal stability, and low N_2_O formation, indicating that simply increasing the total Cu content is not beneficial for improving SCR selectivity.

The combined characterization results reveal that Cu/Al regulation strongly affects the distribution, reducibility, and hydrothermal stability of Cu species in Cu-SSZ-13. H_2_-TPR, UV-vis DRS, XPS, and EPR results indicate that increasing the Cu/Al ratio increases the amount of reducible and EPR-active Cu^2+^ species, but excessive Cu loading also favors the generation of CuO_*x*_-like or less selective Cu environments. Hydrothermal aging further induces Cu-species redistribution and reconstruction, including the transformation of ionic Cu species and the possible formation of CuO_*x*_-like or strongly interacting Cu species. These changes affect the relative distribution among Z[Cu^2+^(OH)]^+^, Z_2_Cu^2+^, and CuO_*x*_-like species required for selective SCR, thereby contributing to the enhanced N_2_O formation observed after aging. *In situ* DRIFTS results further demonstrate that Cu species influence N_2_O formation by regulating the surface reaction behavior of NH_3_ and NO_*x*_-derived intermediates. At 200 °C, high-Cu samples form more pronounced nitrate/nitrite- and NH_4_NO_3_-related intermediates during NH_3_ adsorption, NO + O_2_ adsorption, and transient gas-switching processes. In addition, hydrothermal aging weakens the NH_3_ adsorption/storage capacity, alters the NO_*x*_ activation behavior, and affects the surface reaction between NH_3_ activation and NO_*x*_ reduction. Therefore, N_2_O suppression over Cu-SSZ-13 requires not only sufficient ionic Cu species for the SCR redox cycle, but also the suppression of excessive CuO_*x*_-like species and nitrate/NH_4_NO_3_-related intermediate accumulation.

In summary, maintaining an appropriate relative distribution among Z[Cu^2+^(OH)]^+^, Z_2_Cu^2+^, and CuO_*x*_-like species over Cu-SSZ-13 enables efficient NO_*x*_ reduction while suppressing N_2_O formation.

## Author contributions

Conceptualization, F. F. and J. M.; investigation, J. M., H. L. and H. Z.; writing—review and editing, F. F, J. M. and X. W.; supervision, K. L., and H. W.; project administration, D. Y.; All authors have read and agreed to the published version of the manuscript.

## Conflicts of interest

The authors declare no conflicts of interest. The funders had no role in the design of the study; in the collection, analyses, or interpretation of data; in the writing of the manuscript; or in the decision to publish the results.

## Data Availability

The data that support the findings of this study are available from the corresponding authors upon request.
